# Supplementary material for: XPC–PARP complexes engage the chromatin remodeler ALC1 to catalyze global genome DNA damage repair
Source: Nat Commun. 2022 Aug 13;13:4762. doi: 10.1038/s41467-022-31820-4 (PMC9376112; doi:10.1038/s41467-022-31820-4)
Supplement: Supplementary file 1 — Supplementary Information [file 41467_2022_31820_MOESM1_ESM.pdf]

Supplementary Information

XPC–PARP complexes engage the chromatin remodeler ALC1 to catalyze global genome DNA damage repair

Charlotte Blessing[1,2,#], Katja Apelt[3,#], Diana van den Heuvel[3], Claudia Gonzalez Leal[1,2], Magdalena B. Rother[3], Melanie van der Woude[4], Román González-Prieto[5,6,7], Adi Yifrach[8], Avital Parnas[8], Rashmi G. Shah[9], Tia Tyrsett Kuo[1,2], Daphne E.C. Boer[3], Jin Cai[1,2], Angela Kragten[3], Hyun-Suk Kim[10], Orlando D. Schärer[10,11], Alfred C.O. Vertegaal[5], Girish M. Shah[9], Sheera Adar[8], Hannes Lans[4], Haico van Attikum[3], Andreas G. Ladurner[1,2,12*], and Martijn S. Luijsterburg[3,*]

[1] Biomedical Center (BMC), Physiological Chemistry, Faculty of Medicine, LMU Munich, Planegg-Martinsried, Germany

[2] International Max Planck Research School (IMPRS) for Molecular Life Sciences, Planegg-Martinsried, Germany

[3] Department of Human Genetics, Leiden University Medical Center, Leiden, The Netherlands

[4] Department of Molecular Genetics, Erasmus MC Cancer Institute, Erasmus University Medical Center, Rotterdam, The Netherlands

[5] Department of Cell and Chemical Biology, Leiden University Medical Center, Leiden, The Netherlands

[6] Genome Proteomics Laboratory, Andalusian Center For Molecular Biology and Regenerative Medicine, Seville, Spain

[7] Department of Cell Biology, University of Seville, Seville, Spain

[8] Department of Microbiology and Molecular Genetics, The Institute for Medical Research Israel-Canada, The Faculty of Medicine, The Hebrew University of Jerusalem, Jerusalem, Israel

[9] Laboratory for Skin Cancer Research, CHU-Q: Laval University Hospital Research Centre of Quebec (CHUL site), Quebec City, Canada

[10] Center for Genomic Integrity, Institute for Basic Science, Ulsan, Republic of Korea

[11] Department of Biological Sciences, School of Life Sciences, Ulsan National Institute of Science and Technology, Ulsan, Republic of Korea

[12] Eisbach Bio GmbH, Planegg-Martinsried, Germany

[#] Shared first authors

[*] Corresponding authors: AGL (andreas.ladurner@med.lmu.de), MSL (m.luijsterburg@lumc.nl)

Supplementary Figure 1

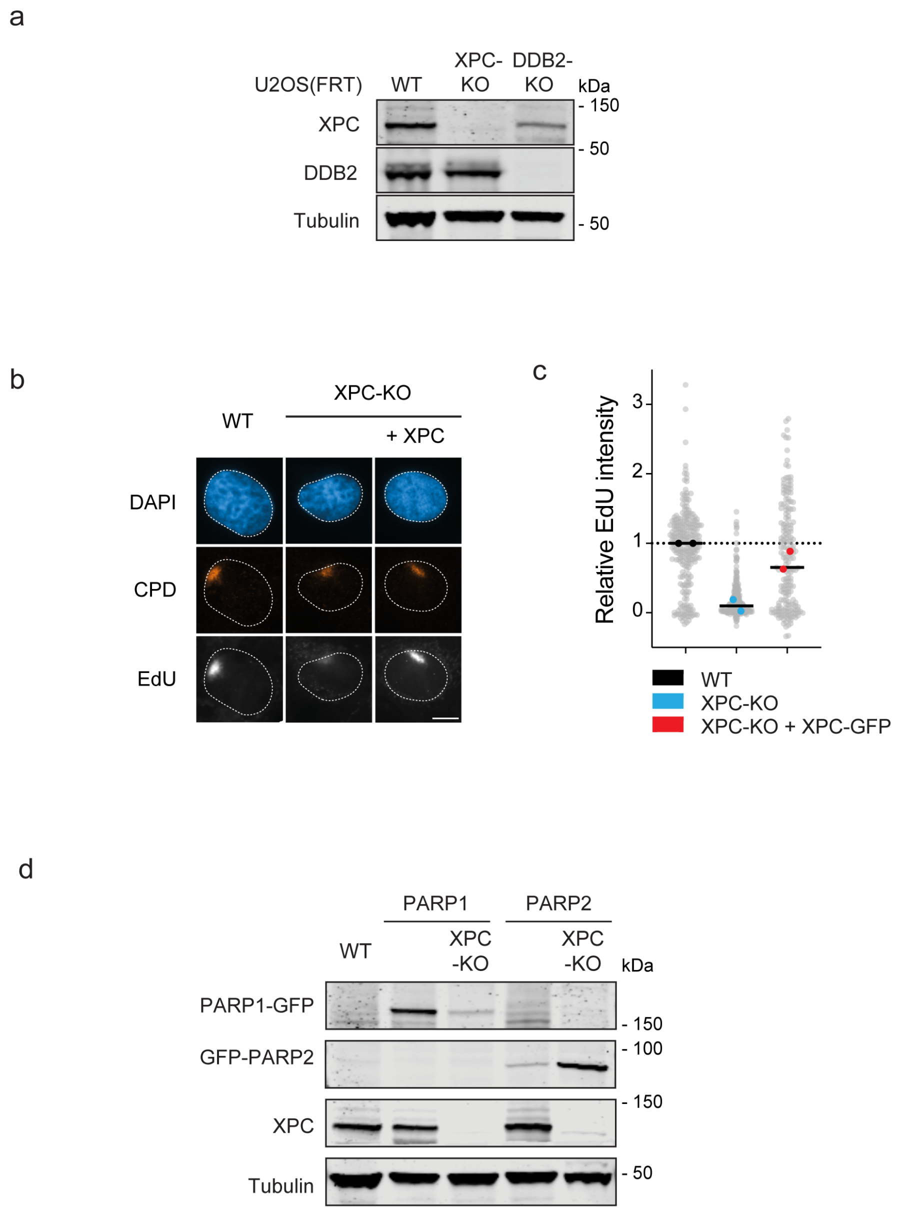

Supplementary Figure 1: Repair defects in XPC-KO cells. (**a**) Western blot of U2OS (FRT) WT, XPC-KO and DDB2-KO cells. Two independent replicates of each IP experiment were performed obtaining similar results. (**b**) Representative images and (**c**) quantification of unscheduled DNA synthesis experiments in U2OS (FRT) WT, XPC-KO, XPC-KO + XPC-GFP cells upon UV-C irradiation. 191-254 cells were analyzed in 2 independent experiments. All cells are depicted as individual data points (grey). The median of each biological replicate is depicted as a colored point, while the bar represents the median of all data points. (**d**) Western blot of U2OS (FRT) WT and XPC-KO cells expressing PARP1-GFP or GFP-PARP2. Two independent replicates of each IP experiment were performed obtaining similar results. The scale bar in (**b**) is 5 µm.

Supplementary Figure 2

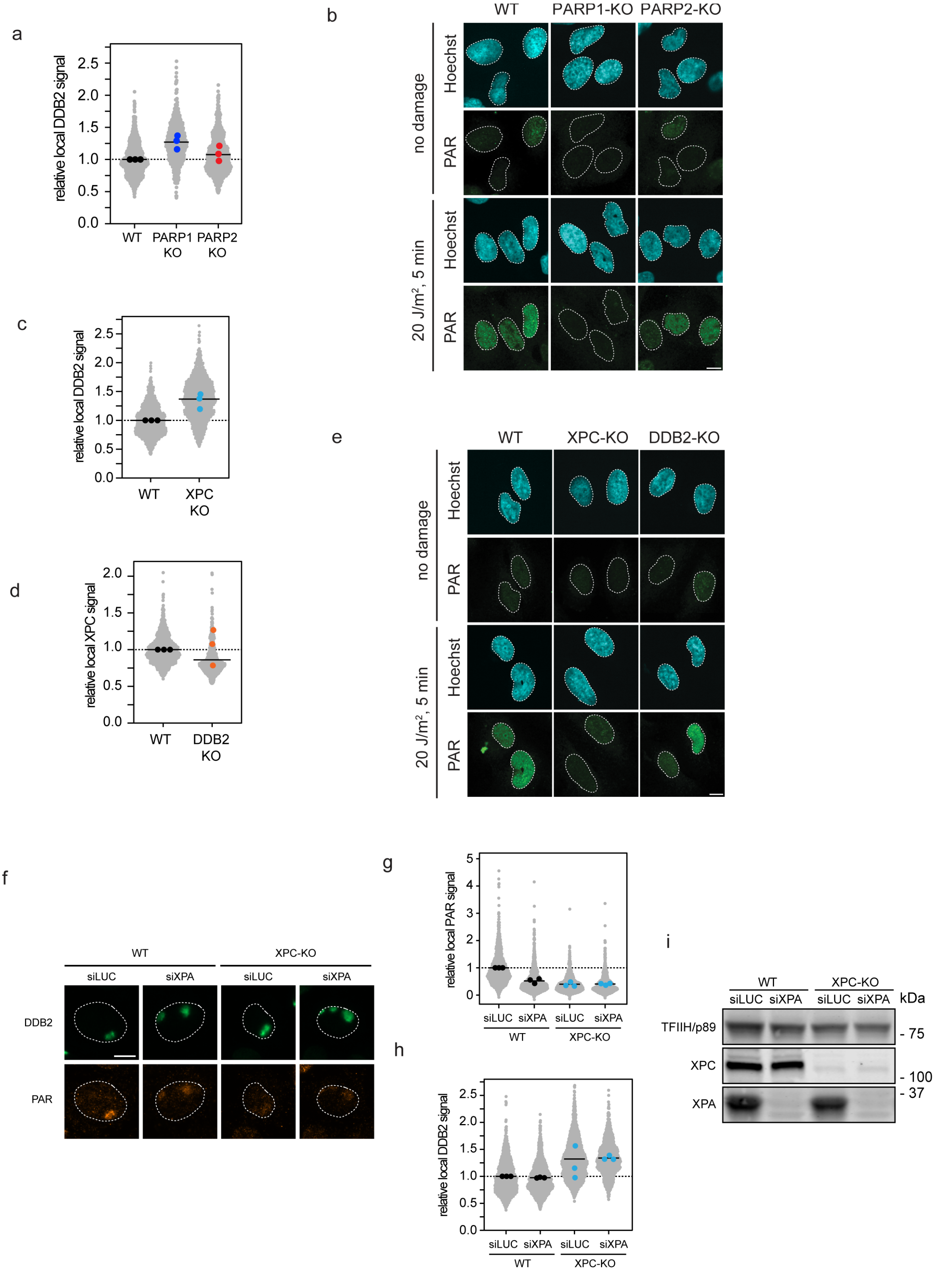

Supplementary Figure 2: Poly-(ADP-ribose) levels at UV lesions. (**a**) Quantification of DDB2 levels 10 minutes after local UV-C irradiation (30 J/m2) by immunofluorescence in U2OS WT, PARP1-KO and PARP2-KO cells shown in **Figure 4a**. >100 cells were analyzed per condition from 3 independent experiments. (**b**) Representative images of poly-(ADP-ribose) (PAR) levels 5 minutes after UV-C irradiation (20 J/m^2) by immunofluorescence (Millipore; MABE1031) in U2OS WT, PARP1-KO and PARP2-KO cells. This experiment was repeated three times obtaining similar results. (**c**) Quantification of DDB2 levels 10 minutes after local UV-C irradiation (30 J/m2) by immunofluorescence in U2OS WT and XPC-KO cells shown in **Figure 4e**. >100 cells were analyzed per condition from 3 independent experiments. >100 cells were analyzed per condition from 3 independent experiments. (**d**) Quantification of XPC levels 10 minutes after local UV-C irradiation (30 J/m2) by immunofluorescence in U2OS WT and DDB2-KO cells shown in **Figure 4g**. >100 cells were analyzed per condition from 3 independent experiments. >100 cells were analyzed per condition from 3 independent experiments. (**e**) Representative images of PAR levels 5 minutes after UV-C irradiation (20 J/m^2) by immunofluorescence (Millipore; MABE1031) in U2OS (FRT) WT, XPC-KO and DDB2-KO cells. This experiment was repeated three times obtaining similar results. (**f**) Representative images and (**g, h**) quantification of poly-(ADP-ribose) (PAR) levels (Trevigen, 4335-MC-100) (**g**) or DDB2 levels (**h**) 10 minutes after local UV-C irradiation (30 J/m^2) by immunofluorescence in U2OS WT or XPC-KO cells transfected with the indicated siRNAs. The median of each biological replicate is depicted as a colored point, while the bar represents the median of all data points. >100 cells were analyzed per condition from 3 independent experiments. (**i**) Western blot of U2OS WT and XPC-KO cells transfected with the indicated siRNAs. Two independent replicates of each western blot were performed obtaining similar results. The scale bar in (**b**, **e**, **f**) is 5 µm.

Supplementary Figure 3

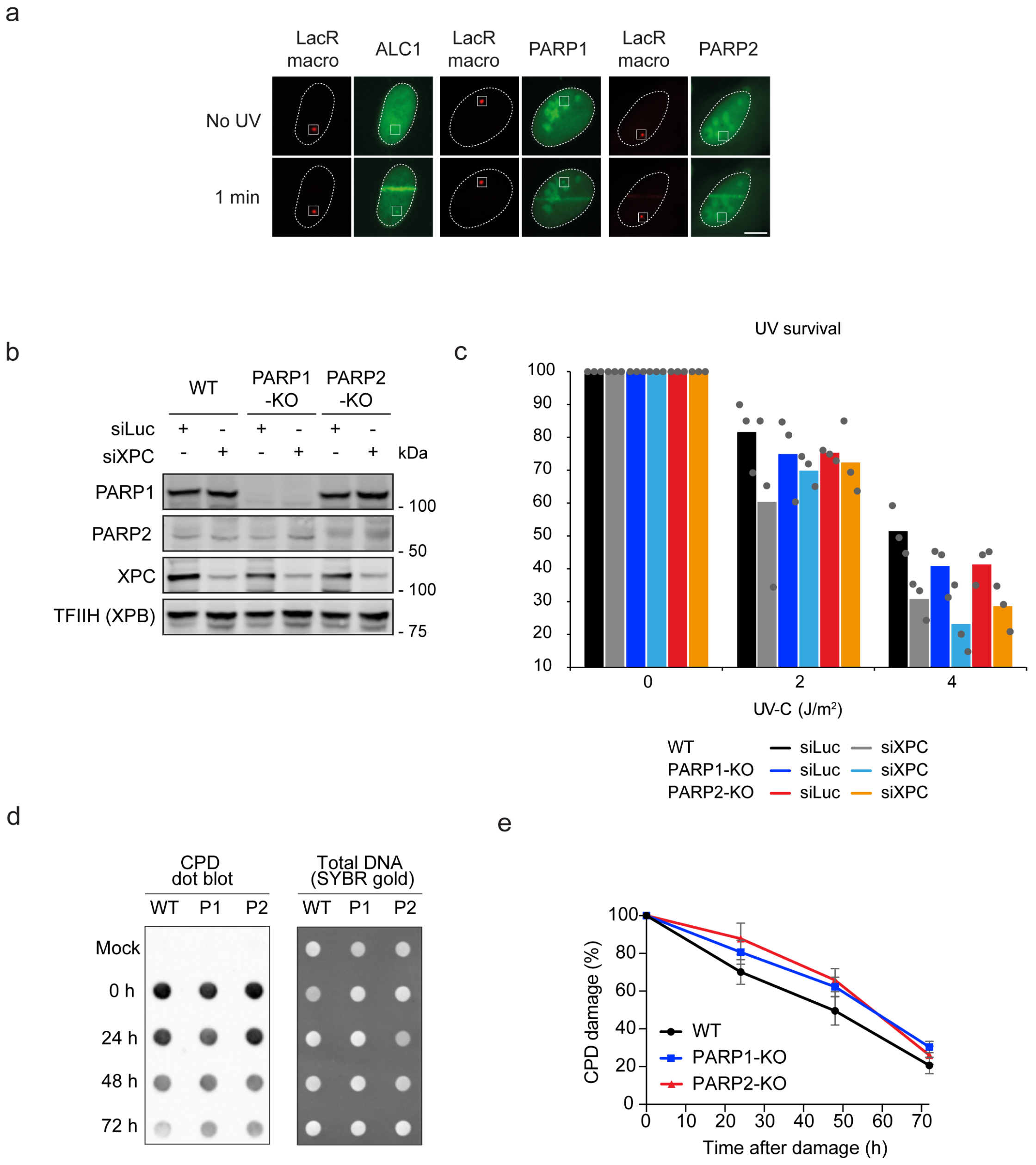

Supplementary Figure 3: CPD repair kinetics in PARP1/2-KO cells. (**a**) Representative images of GFP-tagged ALC1, PARP1 or PARP2 recruitment to the LacO array upon tethering to the indicated mCherry-LacR-macrodomain. Pictures were taken before and 1 min after UV-C micro-irradiation. See **Figure 5e** for additional pictures and quantifications. (**b**) Western blot of U2OS WT, PARP1-KO or PARP2-KO transfected with the indicated siRNAs. Three independent replicates of each western blot were performed obtaining similar results. (**c**) Clonogenic survival assays of U2OS WT, PARP1-KO or PARP2-KO transfected with the indicated siRNAs. The median of each biological replicate is depicted as a grey point, while the colored bar represents the median of 3 independent experiments. (**d**) Representative dot blots and (**e**) quantification of CPD levels in U2OS WT, PARP1-KO and PARP2-KO cells at different time points after UV-C damage (20 J/m^2). The data is depicted as mean + S.E.M. of 4 independent experiments. The scale bar in (**a**) is 5 μm.

Supplementary Figure 4

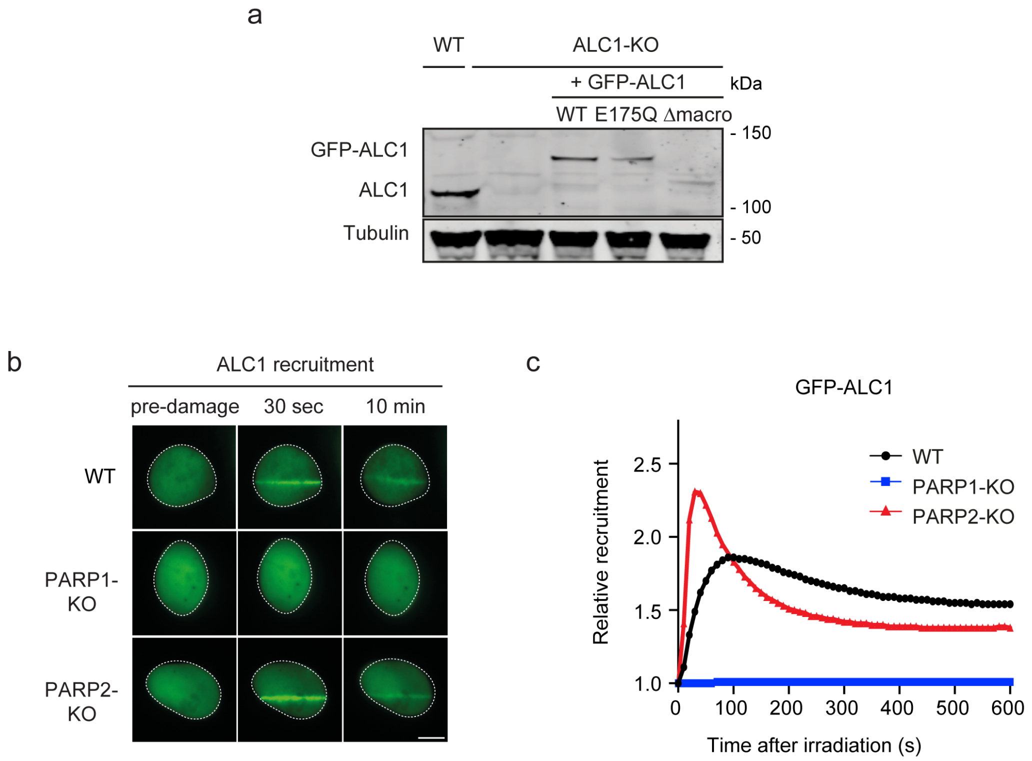

Supplementary Figure 4: Recruitment of ALC1 in PARP1/2-KO cells. (**a**) Western blot of U2OS (FRT) ALC1-KO cells expressing GFP-ALC1 WT, GFP-ALC1 E175Q or GFP-ALC1 ∆macrodomain. Two independent replicates of each western blot were performed obtaining similar results. (**b**) Representative images and (**c**) recruitment kinetics of GFP-ALC1 in U2OS, PARP1-KO and PARP2-KO cells upon UV-C irradiation. 53-60 cells were analyzed from 2 independent experiments (n=2). The data are shown as mean + SEM. The scale bar in (**b**) is 5 µm.

Supplementary Figure 5

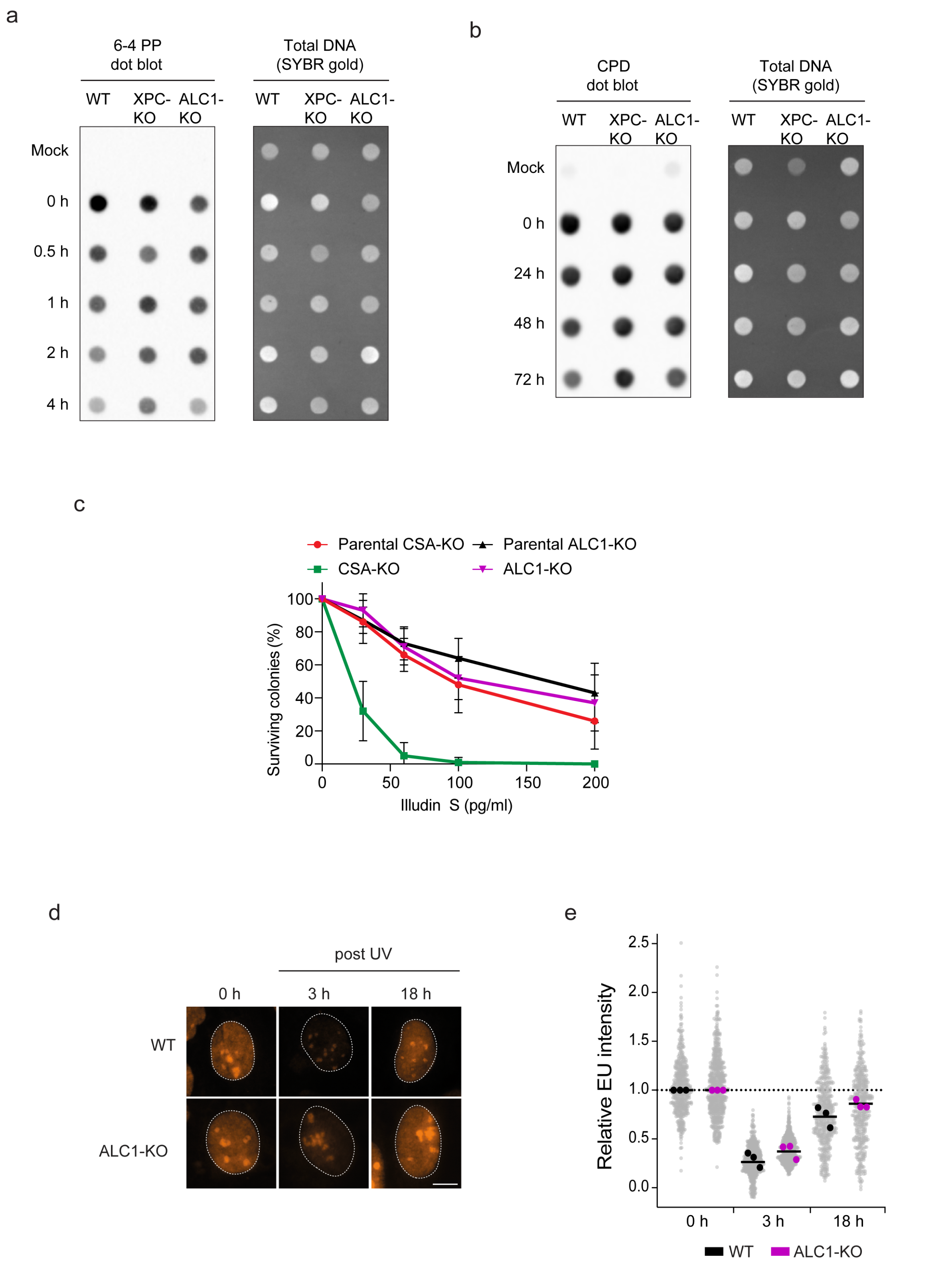

Supplementary Figure 5: Effects of ALC1-KO on nucleotide excision repair. (**a**, **b**) Representative dot blots of 6-4PP (**a**) or CPD (**b**) in U2OS WT, XPC-KO or ALC1-KO. See Figure **8a**, **b** for a quantification. (**c**) Clonogenic survival assays of U2OS ALC1-KO and U2OS (FRT) CSA-KO cells and the respective parental cell lines upon Illudin S treatment. The data is depicted as mean + S.E.M. from 3 independent experiments. (**d**) Representative images and (**e**) quantification of recovery of RNA synthesis assays after UV-C irradiation. 78-212 cells were analyzed in three independent experiments. All cells are depicted as individual data points (grey). The median of each biological replicate is depicted as a colored point, while the bar represents the median of all data points. The scale bar in (**d**) is 5 µm.

Supplementary Figure 6

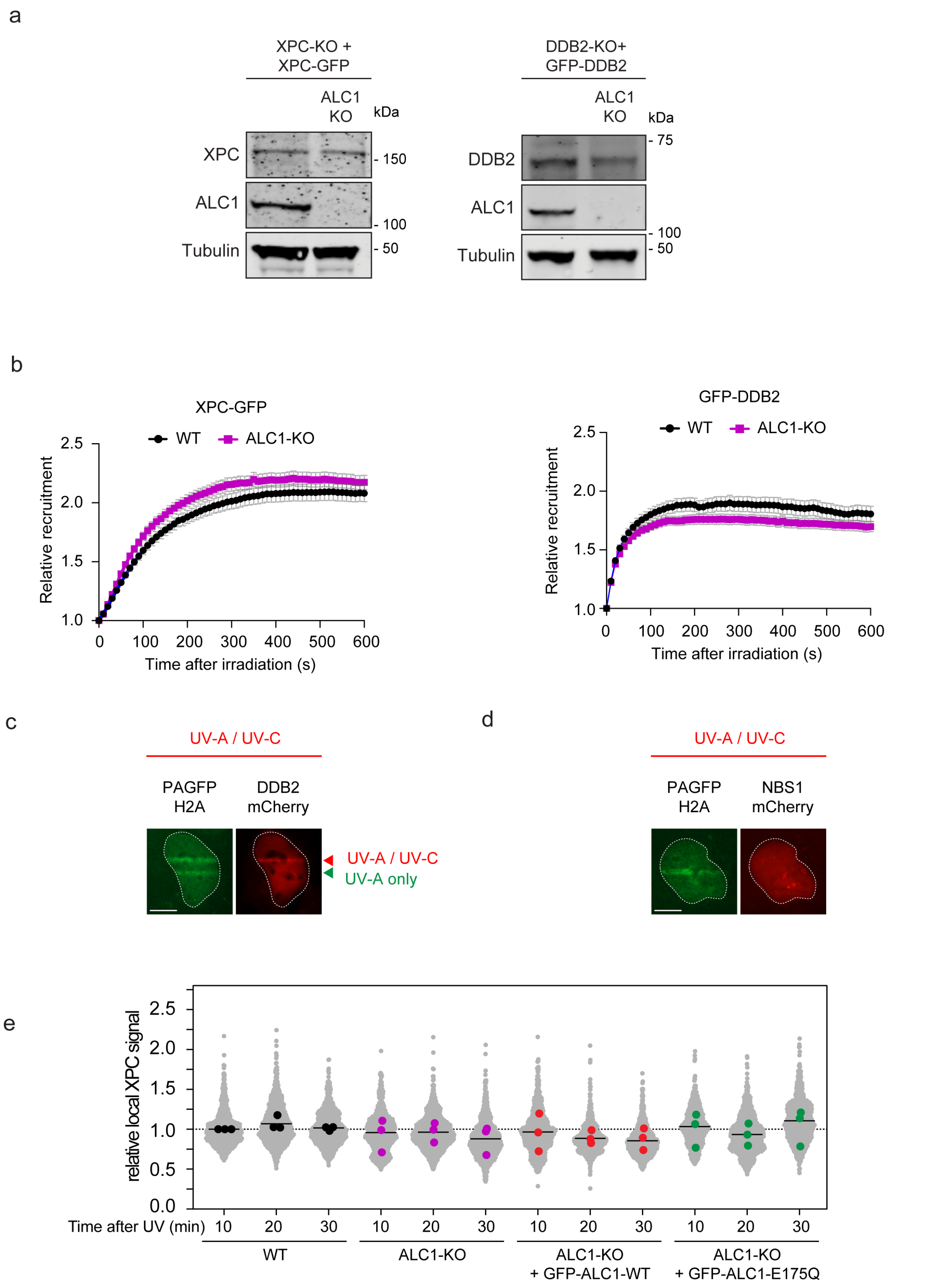

Supplementary Figure 6: Recruitment of XPC and DDB2 in ALC-deficient cells. (**a**) Western blot of U2OS (FRT) XPC-KO + XPC-GFP and XPC ALC1-dKO + XPC-GFP cells, and U2OS (FRT) DDB2-KO + GFP-DDB2 and DDB2 ALC1-dKO + GFP-DDB2 cells. Two independent replicates of each western blot were performed obtaining similar results. (**b**) Recruitment kinetics of XPC-GFP or GFP-DDB2 at sites of local UV-C laser irradiation. 80-135 cells were analyzed in 3 independent experiments. The data are shown as mean + SEM normalized to pre-damage GFP intensity at micro-irradiation sites. (**c**) Representative image of cell expressing PAGFP-H2A and mCherry-DDB2 that has been sequentially irradiated with UV-C and UV-A lasers (upper track) or only with a UV-A laser (lower track). This experiment was performed three times obtaining similar results. (**d**) Representative image of cell expressing PAGFP-H2A and NBS1-mCherry that has been sequentially irradiated with UV-C and UV-A lasers. This experiment was performed three times obtaining similar results. (**e**) Quantification of XPC recruitment 10, 20 and 30 minutes after local UV-C irradiation (30 J/m^2) by immunofluorescence in U2OS WT, ALC1-KO, ALC1-KO + GFP-ALC1, ALC1-KO + GFP-ALC1 E175Q. Quantification of PAR levels in the same cells is shown in **Figure 9d**. The median of each biological replicate is depicted as a colored point, while the bar represents the median of all data points. >80 cells were analyzed per condition from 3 independent experiments. The scale bar in (**c**, **d**) is 5 µm.

Supplementary table 1. Cell lines

Cell lines	Origin
U2OS	Nicholas D Lakin (Ronson et al., 2018)
U2OS 2-6-3	Susan Janicki (Janicki et al., 2004)
U2OS PARP1-KO	Nicholas D Lakin (Ronson et al., 2018)
U2OS PARP2-KO	Nicholas D Lakin (Ronson et al., 2018)
U2OS(FRT)	Daniel Durocher (Panier et al., 2012)
U2OS(FRT) ALC1-KO	This study
U2OS(FRT) ALC1-KO + GFP-ALC1^{E175Q}	This study
U2OS(FRT) ALC1-KO + GFP-ALC1^{WT}	This study
U2OS(FRT) ALC1-KO + GFP-ALC1$^{\Delta MACRO}$	This study
U2OS(FRT) CSA-KO	(van der Weegen et al., 2020)
U2OS(FRT) DDB2 ALC1-dKO + GFP-DDB2	This study
U2OS(FRT) DDB2-KO	This study
U2OS(FRT) DDB2-KO + GFP-DDB2	This study
U2OS(FRT) DDB2-KO + GFP-ALC1	This study
U2OS(FRT) GFP-ALC1	This study
U2OS(FRT) GFP-NLS	Haico van Attikum (Luijsterburg et al., 2017)
U2OS(FRT) PARP1-GFP	This study
U2OS(FRT) GFP-PARP2	This study
U2OS(FRT) XPC ALC1-dKO + XPC-GFP	This study
U2OS(FRT) XPC-KO	This study
U2OS(FRT) XPC-KO + GFP-PARP2	This study
U2OS(FRT) XPC-KO + PARP1-GFP	This study
U2OS(FRT) XPC-KO + XPC-GFP	This study
U2OS(FRT) XPC-KO + GFP-ALC1	This study

Supplementary table 2. Plasmids

Plasmids	Origin
mCherry-LacR-NLS-C1-Macro H2A.1 (184aa-370aa)	This study
mCherry-LacR-NLS-Stop	(van der Weegen et al., 2020)
pcDNA5-FRT-TO-Hygro	Invitrogen
pcDNA5-FRT-TO-Hygro (NheI)	This study
pcDNA5-FRT-TO-Hygro- GFP-ALC1^{E175Q}	(Blessing et al., 2020)
pcDNA5-FRT-TO-Hygro- GFP-ALC1^{WT}	(Blessing et al., 2020)
pcDNA5-FRT-TO-Hygro- GFP-ALC1$^{\Delta MACRO}$	This study
pcDNA5-FRT-TO-Hygro- PARP1-GFP	This study
pcDNA5-FRT-TO-Hygro-GFP-PARP2	This study
pcDNA5-FRT-TO-Puro-GFP-DDB2	This study
pcDNA5-FRT-TO-Puro-GFP-NLS	(Luijsterburg et al., 2017)
pcDNA5-FRT-TO-Puro-XPC-GFP	This study
pDDB2-mCherry	(Luijsterburg et al., 2012)
pEGFP-PARP2	(Blessing et al., 2020)
pNBS1-mCherry	(Luijsterburg et al., 2016)
pOG44	Invitrogen
pPAGFP-H2A	(Luijsterburg et al., 2016)
pPARP1-EGFP	(Mortusewicz et al., 2007)
pX458 (Cas9)	Addgene #48138

Supplementary table 3. Sequences of primers

sgRNAs	Sequence/Origin
macroH2A1.1-GFP-fw	GAATTCTACAGTCCTCTCCACCAAGAGC
macroH2A1.1-GFP-rv	GGATCCTTAGTCCAGCTTGGCCATTTCC
pcDNA5/FRT/TO-Hygro-Nhe-fw	ATCCAGCCTCCGGACGCTAGCGTTTAAAC
pcDNA5/FRT/TO-Hygro-Nhe-rv	AAGTTTAAACGCTAGCGTCCGGAG
pEGFP-PARP2-fw	ATATATGCTAGCATGGTGAGCAAGGGCGAGGAG
pEGFP-PARP2-rv	ATATATGCGGCCGCTCACCACAGCTGAAGGAAATTAAACTG
pPARP1-EGFP-fw	ATATATGCGGCCGCATGGCGGAGTCTTCGGATAAGC
pPARP1-EGFP-rv	ATATATCTCGAGTTACTTGTACAGCTCGTCCATGCC

Supplementary table 4. Sequences of sgRNAs and siRNAs

sgRNAs and siRNAs	Sequence/Origin
sgRNA ALC1	CCATCGGGTTTTACTTTTCTCCC
sgRNA CSA	CAACTTTGTGACTTGAAGTCTGG
sgRNA DDB2	CCTAGCAGAAGATGTGACTCAGA
sgRNA XPC	TGGGGGTTTCTCATCTTCAAAGG
siRNA Luciferase	CGTACGCGGAATACTTCGA
siRNA non-target (NT)	ThermoFisher Silencer Negative Control #1, AM4611
siRNA XPA	CAGAGATGCTGATGATAAA
siRNA XPC	TAGCAAATGGCTTCTATCGAA

Supplementary table 5. Antibodies

Antibodies	Host	Manufacturer	Use	Antibody identifier
6-4PP	Mouse	Cosmo bio, NM-DND-002	Immunoblot: 1:2000	N/A
ALC1	Rabbit	Homemade	WB: 1:1000	aML#144
Alexa 488 anti-rabbit IgG	Goat	Thermo fisher Scientific A-11034	IF: 1:1000	aML#012
Alexa 488 anti-mouse IgG	Goat	Thermo fisher Scientific A-11029	IF: 1:1000	aML#013
Alexa 555 anti-rabbit IgG	Goat	Thermo fisher Scientific A-21429	IF: 1:1000	aML#014
Alexa 555 anti-mouse IgG	Goat	Thermo fisher Scientific A-21424	IF: 1:1000	aML#015
Alexa 555 anti-mouse IgG	Donkey	Thermo fisher Scientific A-31570	IF: 1:1000	aML#171
Alexa 647 anti-rabbit IgG	Goat	Thermo fisher Scientific A-21245	IF: 1:1000	aML#016
Alexa 647 anti-mouse IgG	Goat	Thermo fisher Scientific A-21235	IF: 1:1000	aML#017
Alexa 647 anti-goat IgG	Donkey	Thermo fisher Scientific A32849	IF: 1:1000	aML#176
CF680 anti-rabbit IgG	Goat	Biotium, VWR #20067	WB: 1:10000	aML#010
CF770 anti-mouse IgG	Goat	Biotium, VWR #20077	WB: 1:10000	aML#009
CPD	Mouse	Cosmo Bio (TDM2 clone); CAC-NM-DND-001	IF: 1:1000<br>Immunoblot: 1:4000	N/A
DDB2	Goat	R&D Systems Netherlands; AF3297-SP	WB: 1:1000	aML#107
GFP	Goat	Homemade	WB: 1:2000	N/A
GFP	Mouse	Roche, 11814460001	WB: 1:1000	aML#011
PAR	Mouse	Mouse monoclonal 10H (ascites) (Homemade)	WB: 1:500	N/A
PAR	Mouse	Trevigen, 4335-MC-100	IF: 1:1000	aML#174
PAR-binding reagent	Rabbit	Millipore; MABE1031	IF: 1:500	N/A
PAR-binding reagent	Rabbit	Millipore; MABE1016	WB: 1:1000	N/A
PARP1	Rabbit	Cell signalling; #9542S	WB: 1:1000	aML#060
PARP1	Rabbit	Homemade	WB: 1:10,000	N/A
PARP1	Mouse	C2-10: Enzo: BML-SA250-0050	WB: 1:2000	N/A
PARP2	Mouse	Enzo; clone: 4G8 (ALX-804-639-L001)	WB: 1:200	aML#126
PARP2	Rabbit	Active Motif: Cat# 39743	WB: 1:1000	N/A
Tubulin	Mouse	Sigma; T6199	WB: 1:1000	aML#008
XPA	Rabbit	Gift from Rick Wood (CJ1)	WB: 1 in 10.000	aML#079
XPB (ERCC3, p89)	Mouse	Millipore, MABE1123	WB: 1 in 2000	aML#101
XPC	Rabbit	Novus Biologicals: NB100-58801	WB: 1:1000<br>IF: 1:500	aML#077
XPC	Rabbit	Gene Tex: GTX70309	WB: 1:1000	N/A

Supplementary references

Blessing, C., I.K. Mandemaker, C. Gonzalez-Leal, J. Preisser, A. Schomburg, and A.G. Ladurner. 2020. The Oncogenic Helicase ALC1 Regulates PARP Inhibitor Potency by Trapping PARP2 at DNA Breaks. Mol Cell. 80:862-875 e866.

Janicki, S.M., T. Tsukamoto, S.E. Salghetti, W.P. Tansey, R. Sachidanandam, K.V. Prasanth, T. Ried, Y. Shav-Tal, E. Bertrand, R.H. Singer, and D.L. Spector. 2004. From silencing to gene expression: real-time analysis in single cells. Cell. 116:683-698.

Luijsterburg, M.S., I. de Krijger, W.W. Wiegant, R.G. Shah, G. Smeenk, A.J. de Groot, A. Pines, A.C. Vertegaal, J.J. Jacobs, G.M. Shah, and H. van Attikum. 2016. PARP1 Links CHD2-Mediated Chromatin Expansion and H3.3 Deposition to DNA Repair by Non-homologous End-Joining. Mol Cell. 61:547-562.

Luijsterburg, M.S., M. Lindh, K. Acs, M.G. Vrouwe, A. Pines, H. van Attikum, L.H. Mullenders, and N.P. Dantuma. 2012. DDB2 promotes chromatin decondensation at UV-induced DNA damage. J Cell Biol. 197:267-281.

Luijsterburg, M.S., D. Typas, M.C. Caron, W.W. Wiegant, D. van den Heuvel, R.A. Boonen, A.M. Couturier, L.H. Mullenders, J.Y. Masson, and H. van Attikum. 2017. A PALB2-interacting domain in RNF168 couples homologous recombination to DNA break-induced chromatin ubiquitylation. eLife. 6.

Mortusewicz, O., J.C. Ame, V. Schreiber, and H. Leonhardt. 2007. Feedback-regulated poly(ADP-ribosyl)ation by PARP-1 is required for rapid response to DNA damage in living cells. Nucleic Acids Res.

Panier, S., Y. Ichijima, A. Fradet-Turcotte, C.C. Leung, L. Kaustov, C.H. Arrowsmith, and D. Durocher. 2012. Tandem protein interaction modules organize the ubiquitin-dependent response to DNA double-strand breaks. Mol Cell. 47:383-395.

Ronson, G.E., A.L. Piberger, M.R. Higgs, A.L. Olsen, G.S. Stewart, P.J. McHugh, E. Petermann, and N.D. Lakin. 2018. PARP1 and PARP2 stabilise replication forks at base excision repair intermediates through Fbh1-dependent Rad51 regulation. Nat Commun. 9:746.

van der Weegen, Y., H. Golan-Berman, T.E.T. Mevissen, K. Apelt, R. Gonzalez-Prieto, J. Goedhart, E.E. Heilbrun, A.C.O. Vertegaal, D. van den Heuvel, J.C. Walter, S. Adar, and M.S. Luijsterburg. 2020. The cooperative action of CSB, CSA, and UVSSA target TFIIH to DNA damage-stalled RNA polymerase II. Nat Commun. 11:2104.
