# Supplementary material for: XPC–PARP complexes engage the chromatin remodeler ALC1 to catalyze global genome DNA damage repair
Source: Nat Commun. 2022 Aug 13;13:4762. doi: 10.1038/s41467-022-31820-4 (PMC9376112; doi:10.1038/s41467-022-31820-4)
Supplement: Supplementary file 3 — Reporting Summary [file 41467_2022_31820_MOESM3_ESM.pdf]

nature portfolio

Corresponding author(s): Martijn S. Luijsterburg

Last updated by author(s): Jun 20, 2022

Reporting Summary

Nature Portfolio wishes to improve the reproducibility of the work that we publish. This form provides structure for consistency and transparency in reporting. For further information on Nature Portfolio policies, see our Editorial Policies and the Editorial Policy Checklist.

Statistics

For all statistical analyses, confirm that the following items are present in the figure legend, table legend, main text, or Methods section.

n/a	Confirmed	
☐	☒	The exact sample size (*n*) for each experimental group/condition, given as a discrete number and unit of measurement
☐	☒	A statement on whether measurements were taken from distinct samples or whether the same sample was measured repeatedly
☐	☒	The statistical test(s) used AND whether they are one- or two-sided<br>*Only common tests should be described solely by name; describe more complex techniques in the Methods section.*
☒	☐	A description of all covariates tested
☒	☐	A description of any assumptions or corrections, such as tests of normality and adjustment for multiple comparisons
☐	☒	A full description of the statistical parameters including central tendency (e.g. means) or other basic estimates (e.g. regression coefficient) AND variation (e.g. standard deviation) or associated estimates of uncertainty (e.g. confidence intervals)
☐	☒	For null hypothesis testing, the test statistic (e.g. *F*, *t*, *r*) with confidence intervals, effect sizes, degrees of freedom and *P* value noted<br>*Give P values as exact values whenever suitable.*
☒	☐	For Bayesian analysis, information on the choice of priors and Markov chain Monte Carlo settings
☒	☐	For hierarchical and complex designs, identification of the appropriate level for tests and full reporting of outcomes
☒	☐	Estimates of effect sizes (e.g. Cohen's *d*, Pearson's *r*), indicating how they were calculated

Our web collection on statistics for biologists contains articles on many of the points above.

Software and code

Policy information about availability of computer code

Data collection	Microscopy images were acquired using a Zeiss AxioImager M2 or D2 widefield fluorescence microscope and ZEN 2012 software (blue edition, version 1.1.0.0). Western blot images were acquired using a Odyssey CLx with Image studio lite software (v5.2) or for western blot with ECL a Amersham Imager 680.
Data analysis	Microscopy images were analyzed in Image J (1.48v). Mass spectrometry data was analyzed using MaxQuant software (v1.6.14) and analysis output was further processed in the Perseus (v1.6.14) computational platform and further processed in Microsoft Excel 365 for comprehensive visualization. Graphs were plotted and analyzed using Graphpad Prism 8 (v8.4.2).

For manuscripts utilizing custom algorithms or software that are central to the research but not yet described in published literature, software must be made available to editors and reviewers. We strongly encourage code deposition in a community repository (e.g. GitHub). See the Nature Portfolio guidelines for submitting code & software for further information.

Data

Policy information about availability of data

All manuscripts must include a data availability statement. This statement should provide the following information, where applicable:

- Accession codes, unique identifiers, or web links for publicly available datasets
- A description of any restrictions on data availability
- For clinical datasets or third party data, please ensure that the statement adheres to our policy

Mass spectrometry proteomics data are presented in main Figures 1a, b and Fig 2a, b, c, d and Fig 7a, b, and have been deposited to the ProteomeXchange Consortium via the PRIDE partner repository (https://www.ebi.ac.uk/pride/) with the dataset identifier PXD025226 (Perez-Riverol et al., 2019). The data is also included as Excel in the Source Data file

Field-specific reporting

Please select the one below that is the best fit for your research. If you are not sure, read the appropriate sections before making your selection.

☒ Life sciences ☐ Behavioural & social sciences ☐ Ecological, evolutionary & environmental sciences

For a reference copy of the document with all sections, see nature.com/documents/nr-reporting-summary-flat.pdf

Life sciences study design

All studies must disclose on these points even when the disclosure is negative.

Sample size	No statistical method was used to predetermine sample size. Sample sizes were chosen for the different experimental approaches based on the technical difficulty and throughput of the individual assays, the chosen sample sizes are consistent with previous publications.
Data exclusions	No data was excluded.
Replication	All replication attempts were successful. The number of replicate experiments are indicated in the figure legends of the manuscript and at least two replicates were performed for each individual approach. Most results were confirmed in multiple cell lines and using complementary approaches.<br><br>- Mass spec findings were confirmed by reciprocal immunoprecipitation and western blot analyses<br>- Effects of knock-out of proteins of interest were confirmed by rescue experiments<br>- Global UV irradiation experiments were confirmed by local UV irradiation experiment
Randomization	There was no allocation of test subjects for any experiments, thus randomization was not applicable to our study
Blinding	Data analyses were performed by unbiased software programs/algorithms blinding was therefore not applicable to our study

Reporting for specific materials, systems and methods

We require information from authors about some types of materials, experimental systems and methods used in many studies. Here, indicate whether each material, system or method listed is relevant to your study. If you are not sure if a list item applies to your research, read the appropriate section before selecting a response.

Materials & experimental systems

n/a	Involved in the study	
☐	☒	Antibodies
☐	☒	Eukaryotic cell lines
☒	☐	Palaeontology and archaeology
☐	☒	Animals and other organisms
☒	☐	Human research participants
☒	☐	Clinical data
☒	☐	Dual use research of concern

Methods

n/a	Involved in the study	
☒	☐	ChIP-seq
☒	☐	Flow cytometry
☒	☐	MRI-based neuroimaging

Antibodies

Antibodies used

6-4PP, Mouse, Cosmo bio, NM-DND-002, Immunoblot: 1:2000, N/A
ALC1, Rabbit, Homemade, WB: 1:1000, aML#144
Alexa 488 anti-rabbit IgG, Goat, Thermo fisher Scientific A-11034, IF: 1:1000, aML#012
Alexa 488 anti-mouse IgG, Goat, Thermo fisher Scientific A-11029, IF: 1:1000, aML#013
Alexa 555 anti-rabbit IgG, Goat, Thermo fisher Scientific A-21429, IF: 1:1000, aML#014
Alexa 555 anti-mouse IgG, Goat, Thermo fisher Scientific A-21424, IF: 1:1000, aML#015
Alexa 555 anti-mouse IgG, Donkey, Thermo fisher Scientific A-31570, IF: 1:1000, aML#171
Alexa 647 anti-rabbit IgG, Goat, Thermo fisher Scientific A-21245, IF: 1:1000, aML#016
Alexa 647 anti-mouse IgG, Goat, Thermo fisher Scientific A-21235, IF: 1:1000, aML#017
Alexa 647 anti-goat IgG, Donkey, Thermo fisher Scientific A32849, IF: 1:1000, aML#176
CF680 anti-rabbit IgG Goat, Biotium, VWR #20067, WB: 1:10000, aML#010
CF770 anti-mouse IgG Goat, Biotium, VWR #20077, WB: 1:10000, aML#009
CPD, Mouse, Cosmo Bio (TDM2 clone); CAC-NM-DND-001, IF: 1:1000 Immunoblot: 1:4000, N/A
DDB2, Goat, R&D Systems Netherlands; AF3297-SP, WB: 1:1000, aML#107
GFP, Goat, Homemade, WB: 1:2000, N/A
GFP, Mouse, Roche, 11814460001, WB: 1:1000, aML#011
PAR, Mouse, Mouse monoclonal 10H (ascites) (Homemade), WB: 1:500, N/A
PAR, Mouse, Trevigen, 4335-MC-100, IF: 1:1000, aML#174

PAR-binding reagent, Rabbit, Millipore; MABE1031, IF: 1:500, N/A
PAR-binding reagent, Rabbit, Millipore; MABE1016, WB: 1:1000, N/A
PARP1, Rabbit, Cell signalling; #9542S, WB: 1:1000, aML#060
PARP1, Rabbit, Homemade, WB: 1:10,000, N/A
PARP1, Mouse, C2-10: Enzo: BML-SA250-0050, WB: 1:2000, N/A
PARP2, Mouse, Enzo; clone: 4G8 (ALX-804-639-L001), WB: 1:200, aML#126
PARP2, Rabbit, Active Motif: Cat# 39743, WB: 1:1000, N/A
Tubulin, Mouse, Sigma; T6199, WB: 1:1000, aML#008
XPA, Rabbit, Gift from Rick Wood (CJ1), WB: 1 in 10.000, aML#079
XPB (ERCC3, p89), Mouse, Millipore, MABE1123, WB: 1 in 2000, aML#101
XPC, Rabbit, Novus Biologicals: NB100-58801, WB: 1:1000 IF: 1:500, aML#077
XPC, Rabbit, Gene Tex: GTX70309, WB: 1:1000, N/A

Validation

The following antibodies were validated in knockout cells:

ALC1, Rabbit Homemade WB: 1:1000 aML#144
DDB2, Goat R&D Systems Netherlands; AF3297-SP WB: 1:1000 aML#107
PARP1, Rabbit Cell signalling; #9542S WB: 1:1000 aML#060
PARP1, Rabbit Homemade WB: 1:10,000 N/A
PARP1, Mouse C2-10: Enzo: BML-SA250-0050 WB: 1:2000 N/A
PARP2, Mouse Enzo; clone: 4G8 (ALX-804-639-L001) WB: 1:200 aML#126
PARP2, Rabbit Active Motif: Cat# 39743 WB: 1:1000 N/A
XPA, Rabbit Gift from Rick Wood (CJ1) WB: 1 in 10.000 aML#079
XPC, Rabbit Novus Biologicals: NB100-58801 WB: 1:1000, IF: 1:500 aML#077
XPC, Rabbit Gene Tex: GTX70309 WB: 1:1000 N/A

The following antibodies showed a reduced signal in PARP1-KO cells demonstrating specificity

PAR, Mouse Homemade (10H) WB: 1:500 N/A
PAR, Mouse Trevigen, 4335-MC-100 IF: 1:1000 aML#174
Poly-ADP-ribose binding reagent, Rabbit Millipore; MABE1031 IF: 1:500 N/A

UV-specific staining that disappears with expected kinetics and persists in XPC-KO cells

6-4PP, Mouse Cosmo bio, NM-DND-002 Immunoblot: 1:2000 N/A
CPD, Mouse Cosmo Bio (TDM2 clone); CAC-NM-DND-001 IF: 1:1000, Immunoblot: 1:4000 N/A

The following antibodies were validated in Co-IP experiments:

XPB, (ERCC3, p89) Mouse Millipore, MABE1123 WB: 1 in 2000 aML#101
GFP, Goat Homemade WB: 1:2000 N/A
GFP, Mouse Roche, 11814460001 WB: 1:1000 aML#011

This antibody is a commonly used loading control:

Tubulin, Mouse Sigma; T6199 WB: 1:1000 aML#008

Eukaryotic cell lines

Policy information about cell lines

Cell line source(s)

U2OS Nicholas D Lakin (Ronson et al., 2018)
U2OS 2-6-3 Susan Janicki (Janicki et al., 2004)
U2OS PARP1-KO Nicholas D Lakin (Ronson et al., 2018)
U2OS PARP2-KO Nicholas D Lakin (Ronson et al., 2018)
U2OS(FRT) Daniel Durocher (Panier et al., 2012)
U2OS(FRT) ALC1-KO This study
U2OS(FRT) ALC1-KO + GFP-ALC1E175Q This study
U2OS(FRT) ALC1-KO + GFP-ALC1WT This study
U2OS(FRT) ALC1-KO + GFP-ALC1ΔMACRO This study
U2OS(FRT) CSA-KO (van der Weegen et al., 2020)
U2OS(FRT) DDB2 ALC1-dKO + GFP-DDB2 This study
U2OS(FRT) DDB2-KO This study
U2OS(FRT) DDB2-KO + GFP-DDB2 This study
U2OS(FRT) DDB2-KO + GFP-ALC1 This study
U2OS(FRT) GFP-ALC1 This study
U2OS(FRT) GFP-NLS Haico van Attikum (Luijsterburg et al., 2017)
U2OS(FRT) PARP1-GFP This study
U2OS(FRT) GFP-PARP2 This study
U2OS(FRT) XPC ALC1-dKO + XPC-GFP This study
U2OS(FRT) XPC-KO This study
U2OS(FRT) XPC-KO + GFP-PARP2 This study
U2OS(FRT) XPC-KO + PARP1-GFP This study
U2OS(FRT) XPC-KO + XPC-GFP This study

	U2OS(FRT) XPC-KO + GFP-ALC1 This study
Authentication	Cells were authenticated by STR profiling. All knockout cells were validated by Western blot analysis and DNA sequencing
Mycoplasma contamination	All cell lines were routinely tested for mycoplasma and were nested negative
Commonly misidentified lines (See ICLAC register)	No commonly misidentified cell lines were used in this study

Animals and other organisms

Policy information about studies involving animals; ARRIVE guidelines recommended for reporting animal research

Laboratory animals	C. elegans strains wild type Bristol B2 and animals carrying alleles xpc-1 (tm3886), parp-1 (ok988) and parp-2 (ok344)
Wild animals	This study did not involve wild animals
Field-collected samples	This study did not involve samples collected from the field
Ethics oversight	No ethics oversight is required for studies using C. elegans

Note that full information on the approval of the study protocol must also be provided in the manuscript.
